# Simulated patient contributions to enhancing exercise physiology student clinical assessment skills

**DOI:** 10.1186/s41077-019-0097-6

**Published:** 2019-12-20

**Authors:** Nathan E. Reeves, Monique C. Waite, Neil Tuttle, Andrea Bialocerkowski

**Affiliations:** 1 School of Allied Health Sciences, Gold Coast Campus, Griffith University, Southport, QLD 4222 Australia; 2 School of Health and Rehabilitation Sciences, The University of Queensland, Brisbane, QLD 4222 Australia; 3 Menzies Health Institute Queensland and School of Allied Health Sciences, Griffith University, Southport, QLD 4222 Australia

**Keywords:** Exercise physiology, Simulated-based learning, Simulated patient

## Abstract

**Background:**

The aim of this study was to evaluate exercise physiology students’ perceptions of two simulation-based learning modules focused on communication and interpersonal skills during history taking.

**Methods:**

A prospective, repeated-measures cohort study was conducted with 15 participants. The study evaluated two simulation-based learning modules in a 1-year Graduate Diploma of Exercise Science program. Surveys were administered at four time points: prior to each module and following each module. Students rated their confidence in communication and history taking, and perception of preparedness for practice, motivation for learning, and benefits of undertaking simulation-based learning. Quantitative data were analyzed descriptively and by using repeated measures tests. Qualitative data underwent thematic analyses.

**Results:**

Students reported a significant improvement in their confidence in communication (*P* = 0.043) and in two parameters related to history taking (*P* = 0.034 and 0.035) following the completion of the two modules. There was 96% agreement that the simulation-based learning better prepared students for practice as an exercise physiologist. Significant changes occurred in all aspects of motivation for learning (*P* ranging from < 0.001 to 0.036) except for usefulness, where there was a ceiling effect (medians of 7 on a 7-point scale). Qualitative analysis demonstrated benefit to participants around themes of experiential learning, realism, opportunity to develop clinical skills, and debriefing. Students also made suggestions with respect to the activity structure of the simulation-based learning modules.

**Conclusions:**

The results of this study indicated that simulation-based learning employing SPs increased the confidence and preparedness of exercise physiology students for conducting history taking, a requisite exercise physiology skill. Future studies should include behavioral measures of skill attainment and include follow-up evaluation to appraise the application of these skills into clinical practice.

**Electronic supplementary material:**

The online version of this article (10.1186/s41077-019-0097-6) contains supplementary material, which is available to authorized users.

## Background

In health care, the procedure of verbally gathering information from the patient forms an important part of the initial examination [1]. This process is variably described by health professionals as history taking, subjective assessment, subjective examination, and patient interviewing. This paper uses the term history taking, a process that facilitates the clinician to gather important medical and non-medical information, to support key decisions for diagnosis and management. Previous studies of physicians have demonstrated that history taking alone may accurately inform diagnosis in about 75% of cases [2], and therefore, it is an important skill for all health care professionals. Effective history taking relies on the development of a therapeutic relationship with the patient, which in turn is developed through the use of effective communication and interpersonal skills [3]. Therefore, when teaching students history taking, it is important not only to teach the theoretical aspects of history taking, but also to allow students to practice history taking in a supported learning environment to develop high levels of communication and interpersonal skills.

This paper focuses on the university education of students in the allied health profession of exercise physiology. In Australia, accredited exercise physiologists are recognized allied health professionals who specialize in prescribing clinical exercise interventions for patients with a broad range of pathological populations. These interventions include health and physical activity education, advice and support, and lifestyle modification with a strong focus on achieving behavioral change [4]. As exercise physiologist treatment is patient-centered, skilled history taking is essential to ensure appropriate management.

Becoming an exercise physiologist in Australia involves completing a bachelor or bachelor plus post-graduate degree. Students are taught theory and practice in the context of healthy populations before being introduced to clinical theory and practicum. Until recently, exercise physiology students commenced practicum with theoretical knowledge of history taking but with little or no practical experience. Anecdotal feedback from clinical educators has identified that students entering their first placement lack experience and skill in areas such as history taking, communication, and patient interaction. This perception is supported by a survey of Australian exercise physiology placement supervisors, which identified students as being unprepared, or as having insufficient pre-requisite knowledge or skills, which restricted their willingness to supervise students on practicum [5]. This highlights the difficulties associated with students transferring theory into practice and the impact this has on sourcing clinical placements, a vital accreditation requirement for exercise physiology.

Potentially, the gap between theory and practice could be bridged by the use of simulation-based learning environments (SLEs) [6]. Evidence demonstrates that SLEs are effective in providing university students with authentic learning experiences and can be aimed at developing a range of clinical competencies in a safe and supportive environment [7–10]. Within a SLE, simulated patients (SPs) are well people who are trained to portray patients with common clinical conditions. Students may assess and treat SPs thus applying their knowledge and practicing a variety of clinical skills, including history taking [11]. To date, the literature on working with SPs for developing competency in communication (including history taking) and clinical skills is mainly derived from the fields of nursing and medicine [6, 12–14], physiotherapy [15, 16], and pharmacy [17, 18]. Emerging evidence from a range of other allied health professions also demonstrates trends in effectiveness of SLEs [19, 20].

A systematic review has not been conducted; however, to the authors’ best knowledge, to date, only two studies have investigated the impact of SLEs on exercise physiology student education [21, 22]. Hecimovich and Volet compared the learning gained employing formally trained SPs in a SLE to that of peer patient learning (peers acting as patients) in the assessment and treatment of the musculoskeletal condition, rotator cuff tendinopathy/subacromial impingement syndrome. The results obtained by means of open-ended questions posed to the SPs and peer patients after the simulation suggested that students who treated a SP developed higher levels of clinical skills than those who treated a peer patient, and both groups demonstrated significant gains in skill confidence, knowledge, and motivation for learning. Furthermore, students in the SP group perceived that the SLE positively impacted on their preparation for clinical practice. The authors concluded that there was value in working with SPs to enhance the assessment skills of exercise physiology students in the area of musculoskeletal rehabilitation. While this study demonstrated that the SLE fosters skill development in exercise physiology students, it was focused on skill development in clinical assessment and management of a specific musculoskeletal pathology. The effects of this program on students’ communication and history taking skills are unknown, and therefore, the links between these skills and confidence and preparedness for practice remain unknown.

A more recent study has investigated the contributions of an SLE focusing on interaction with older adults in primary healthcare settings, to exercise physiology student learning on clinical placement [22]. The SPs that participated were volunteer “expert patients,” who were relatively healthy individuals managing their own healthcare portraying themselves*,* rather than being trained to portray a particular clinical case. Following three introductory workshops, the 10 Master of Clinical Exercise Physiology students participated in up to four SLE-based placements. SLE placements included 60–90 min consultation interviews with the expert patients, where students conducted a detailed case history and clinical assessments. Results of the content analysis of student, staff, and expert patient interviews and reflections revealed that the simulation-based education activity was able to achieve its objectives overall, with students reporting improved confidence in communication and clinical skills*.* Expert patients also reported enjoyment and benefit from activity, and clinical supervisors noted improvements in student attitude, knowledge, and skills*.* Interestingly, the authors also reported that some students did not seem to value communicating with the expert patients (e.g., by expressing a preference for the more practical clinical tasks) [22]*.* Further research is therefore needed to investigate how these interpersonal skills may be addressed in simulation-based learning.

Building upon the overall positive results of these previous studies [21, 22], the aim of this research was to evaluate exercise physiology students’ perceptions of two simulation-based learning modules focused on communication and interpersonal skills during history taking. The research question we sought to answer was whether or not the simulation-based learning modules would improve students’ self-confidence in their clinical skills and affect how prepared they felt for clinical practice? A secondary question was what aspects would influence students’ perceptions of the value of the simulation-based learning activities?

## Methods

### Trial design

This study employed a prospective, repeated-measures cohort study design to address the study aim and focused on modules one and two of the suite of five simulation modules completed by students across the year. This study received ethical approval from the university’s Human Research Ethics Committee. Students consented to participation in the research component of the simulation activity (GU Ref No: PES/40/12/HREC).

### Participants

Participants in this study [*n* = 15; entire cohort] were post-graduate students who had completed a 3-year undergraduate degree in exercise science and were enrolled in the Graduate Diploma of Exercise Science program, at one Australian university. All students enrolled in the program were invited to participate in this study via an organization site on the learning and teaching platform. Consent to participate in this educational research was sort and given as part of the pre-simulation survey.

### Activity and setting

**Fig. 1:**
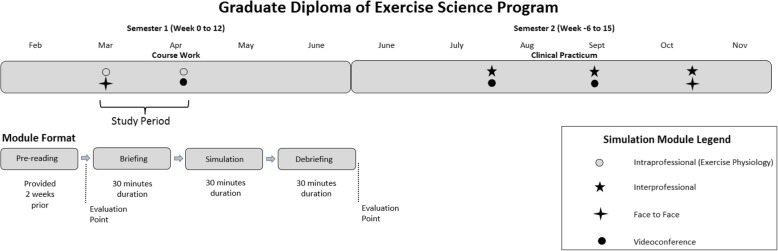
Simulation modules delivered as part of the 1-year exercise physiology program

The simulation-based learning activities implemented were two of a series of five modules embedded in a 1-year Graduate Diploma of Exercise Science program (Fig. 1). Learning activities were themed on history taking, with learning objectives supporting skill transition from theory to practice. Modules 1 and 2 were scheduled in the first semester of the program, prior to the students commencing their clinical practicum. Subsequent modules included the use of videoconferencing to enable access to the SLE while the students were on practicum. Videoconferencing was incorporated in module 2 to prepare students for this delivery mode. The two simulation modules used for this study (See Fig. 1) provided students with the opportunity to develop skills in history taking on a SP in the key pathology areas exercise physiologists encounter in practice: musculoskeletal and neurological conditions. Each module consisted of a 60-min pre-reading (completed by students within the 2 weeks leading up to the simulation day), 30-min briefing (simulation facilitator-led student preparation), 30-min simulation (patient consultation undertaken by the student), and 30-min debrief (simulation facilitator led student and simulated patient reflection). The pre-reading for each module outlined the theoretical underpinnings of best practice for initial assessments, including history taking, introduced the case study scenario, and included a video recording of an experienced Accredited Exercise Physiologist conducting an initial assessment of the “real” patient on which the scenarios were based. Students were grouped into pairs for the activity. In the first simulation module, student A completed the history taking, while student B observed and recorded the feedback. Student roles were then reversed in the second simulation. A simulation facilitator observed both simulated activities and initiated the debriefing phase immediately following the each of the simulation activities. The debriefing included self-reflection on performance, discussion of the case, and feedback from the facilitator, SP, and peer observer. Additional file 1 contains sample questions from a Student Workbook that was provided to students and simulation facilitators prior to the simulation-based learning activity. The questions and prompts were used as a guide to structure the briefing and debriefing sections of the learning activity.

The simulation modules were hosted on the university campus. Clinical consultation rooms and the adjoining waiting area in the Griffith University Health Center were used to create an authentic clinical environment for the simulation. In the first module, students conducted the history taking face-to-face with the SP. For the second module, the web-based videoconferencing platform WebEx® (Cisco WebEx, Milpitas, CA) was used to simulate a telehealth consultation. For this module, the students and SPs were located in adjoining clinic rooms and did not have direct face-to-face contact. All briefing and debriefing activities took place face-to-face in designated adjoining rooms in the Griffith University Health Clinic.

### Simulation facilitators and SPs

The two facilitators who participated in this study were experienced and practicing Accredited Exercise Physiologists, who were trained in simulation facilitation. Facilitator briefing and debriefing sessions were coordinated by the lead author immediately before and after the learning activities. The two SPs, who were also trained, professional actors, had extensive experience in a range of medical and allied health simulations. Prior to the simulation day, the SPs were briefed on the patient profile, learning outcomes of the simulation, and conduct/timing of the simulation-based learning activities. A SP briefing session also occurred immediately before the learning activity commenced which provided SPs with an opportunity for any last minute clarifications.

### Outcome measures

A custom-designed evaluation was administered via iPad surveys at four time points (Table 1). The surveys comprised of published and custom-designed measures of student perceptions according to the learning and reaction levels of Kirkpatrick’s evaluation of training programs [23].

**Table 1:** Simulation module evaluation timings

Outcome measures	Module 1		Module 2	
	Pre	Post	Pre	Post
Self-confidence in clinical skills	●	●	●	●
Perceived impact on preparedness for clinical practice		●		●
Intrinsic motivation inventory	●	●	●	●
Perceptions of specific elements of the simulated learning		●		●

The primary outcomes were students’ self-rated confidence in communication and history taking and perception of preparedness for practice. The “Self-confidence in Clinical Skills” questionnaire was adapted from a tool previously used and found to be reliable in studies of simulation-based learning in physiotherapy education [7, 10]. The original questionnaire comprised of 13 items used to measure students’ confidence in communication, assessment, and management. For this study, the questionnaire was adapted to include 12 questions to align with the learning outcomes associated with the simulation-based learning activities. All items were rated on a 5-point Likert scale (where 1 = strongly disagree and 5 = strongly agree) in response to statements introduced by “I feel confident in my ability to….” The 10 communication skills items were averaged to determine a single score for communication skills. The internal consistency of this revised scale was 0.88, indicating good reliability [24]. The remaining two items were scored separately. Perceived impact on preparedness for clinical practice was measured following each module, on a 5-point Likert scale, where 1 = strongly disagree and 5 = strongly agree, in response to the statement, “The simulation education has made me better prepared to assess and manage a patient with a musculoskeletal (module 1) / neuromuscular (module 2) disorder.”

Secondary outcome measures included student motivation for learning and their perception of the benefits of simulation-based learning. The Intrinsic Motivation Inventory (IMI) [25] (administered pre- and post-modules 1 and 2) was used to measure learners’ motivation for undertaking a learning intervention. Previous studies have demonstrated the IMI has appropriate reliability and validity [26–28]. The IMI version used for the current study consisted of 39 items, with combined means used to determine the following six subscales: interest-enjoyment, perceived competence, pressure-tension, value-usefulness for the development of communication skills, value-usefulness for the development of assessment skills, and value-usefulness for the development of management skills. The items were rated on a 7-point Likert scale in response to truthfulness of the statements (1 = not at all, 4 = somewhat, 7 = very true). To measure students’ perception of the simulation-based learning before undertaking the modules and measure if perceptions changed across the modules, the wording of the pre-simulation version of the IMI was modified to the future tense. For example, “This activity was fun to do” was changed to “This activity will be fun to do.”

An additional set of questions was administered following each module to determine students’ perceptions of specific elements of the simulation-based learning activities (Table 2). Six statements were developed to gather information on the value of working with peers and a SP and the value of simulation-based learning as an educational method. These were rated on a 5-point Likert scale, where 1 = strongly disagree and 5 = strongly agree. The final two items were open-ended questions eliciting students’ perceptions of the most and least effective parts of the simulation-based learning experience.

**Table 2:** Counts, frequencies, and mean (IQR) for ratings of the benefits of simulation-based learning and comparison of post-module 1 and post-module 2 (Wilcoxon signed-ranks, *n* = 14)

Item	Post-module 1					Post-module 2					Comparison	
	% disagree (*n*)	% unsure (*n*)	% agree (*n*)	% strongly agree (*n*)	Median (IQR)	% disagree (*n*)	% unsure (*n*)	% agree (n)	% strongly Agree	Median (IQR)	*Z*	*P*
I found that working with peers on the same SP helped my learning	0.0% (0)	7.1% (1)	21.4% (3)	71.4% (10)	5.00 (4.00–5.00)	0.0% (0)	7.1% (1)	42.9% (6)	50.0% (7)	4.50 (4.00–5.00)	− 1.134	0.257
I was less concerned about making a mistake with SPs than with a real patient	21.4% (3)	7.1% (1)	21.4% (3)	50.0% (7)	4.50 (2.75–5.00)	7.1% (1)	0.0% (0)	50.0% (7)	42.9% (6)	4.00 (4.00–5.00)	− 1.043	0.297
Feedback from a “patient perspective” from role play actors helped my learning	0.0% (0)	21.4% (3)	21.4% (3)	57.1% (8)	5.00 (3.75–5.00)	0.0% (0)	7.1% (1)	21.4% (3)	71.4% (10)	5.00 (4.00–5.00)	− 0.893	0.372
The clinical facilitator was able to give more “frank and honest” feedback in the presence of a SP, compared with a real patient	0.0% (0)	0.0% (0)	42.9% (6)	57.1% (8)	5.00 (4.00–5.00)	7.1% (1)	0.0% (0)	21.4% (3)	71.4% (10)	5.00 (4.00–5.00)	0.000	1.000
This model of education met my learning style	0.0% (0)	7.1% (1)	28.6% (4)	64.3% (9)	5.00 (4.00–5.00)	0.0% (0)	7.1% (1)	28.6% (4)	64.3% (9)	5.00 (4.00–5.00)	0.000	1.000
Simulated learning provides a link between theoretical and practical training	0.0% (0)	0.0% (0)	7.1% (1)	92.9% (13)	5.00 (5.00–5.00)	0.0% (0)	0.0% (0)	28.6% (4)	71.4% (10)	5.00 (4.00–5.00)	− 1.342	0.180

**“**Strongly disagree” omitted as there were no ratings at both time points

IQR indicates interquartile range (quartile 1–quartile 3)

*SP* simulated patient

### Analyses

Repeated measures tests were used to determine change in confidence and motivation across time. Prior to these analyses, tests of normality were undertaken on each variable. Repeated measures ANOVAS with post hoc testing (Bonferroni correction with 95% CI of the differences in means) or the non-parametric equivalent (Friedman test with post hoc Wilcoxon signed-rank tests) were conducted to determine a change in the primary and secondary variables over time. Post hoc testing involved planned contrasts to determine the effect of time. Descriptive statistics were used to summarize students’ preparedness for clinical practice, the perceptions and benefits of simulation-based learning items. A comparison of responses between the two modules was made using the Wilcoxon signed-ranks test. All statistical analyses were conducted with IBM SPSS Statistics, Version 22 Software. The significance level for all analyses was set at *P* < 0.05.

The two open-ended questions were analyzed using qualitative content analysis [29] to identify themes associated with the most and least effective aspects of the simulation-based learning experience. As responses to these questions were similar across the two modules, the responses for module 1 and 2 were pooled. Authors 1 and 2 independently coded participant responses, grouped responses into categories, and categories into themes*.* Following this, they met to discuss their findings to gain consensus on themes*.*

## Results

Fourteen of the 15 (93%) students attended both module 1 and 2, including 6 females and 8 males. Their mean age was 22.1 years (SD = 2.1; range = 20 to 27 years). The majority of participants reported no prior experience with simulation-based education (*n* = 12, 86%) or videoconferencing (*n* = 13, 93%). One student (male, aged 43 years) did not attend the second session. His data were excluded from the analyses. Of the 14 participants, there was a 100% response rate for all questionnaires.

### Change in confidence

There was significant change in confidence in communication across the four time points (*F* = 3.61; *P* = 0.043). Post hoc tests revealed that mean confidence in communication skills increased significantly between pre- and post-module 1 (3.81 ± 0.36 vs 4.13 ± 0.47; *P* = 0.007; 95% CI, 0.11–0.54), as well as between pre- and post-module 2 (3.92 ± 0.63 vs 4.24 ± 0.61; *P* = 0.013; 95% CI, 0.08–0.55). The increase in confidence between pre-module 1 and post-module 2 was also significant (*P* = 0.024; 95% CI, 0.07–0.79).

As shown in Table 2, students reported significant changes in their confidence, particularly in their ability to take a history and to identify clinical information sufficient to make a primary hypothesis about the underlying problem. Post hoc testing revealed a significant increase in confidence on both items from pre to post each module, as well as from pre-module 1 to post-module 2 (Table 3).

**Table 3:** Median (IQR) ratings and results of Friedman’s tests and post hoc comparisons for the assessment confidence parameters recorded pre-module 1, post-module 1, pre-module 2, and post-module 2 (*n* = 14) (where 1 = strongly disagree and 5 = strongly agree)

Item	Module 1		Module 2		Time main effect		Post hoc contrasts		
	Pre (IQR)	Post (IQR)	Pre (IQR)	Post (IQR)	*χ*^2^	*P*	Pre 1–post 2 *P*	Pre 1–post 1 *P*	Pre 2–post 2 *P*
Conduct an effective patient or parent interview (subjective examination)	3.50 (3.00–4.00)	4.00 (3.00–4.25)	4.00 (3.00–4.00)	4.00 (3.75–5.00)	10.481	0.015^a^	0.007^a^	0.034^a^	0.034^a^
Identify clinical information sufficient to make a primary hypothesis about the underlying problem	3.00 (3.00–4.00)	4.00 (3.75–4.00)	4.00 (3.00–4.25)	4.00 (4.00–5.00)	16.161	0.001^a^	0.004^a^	0.007^a^	0.035^a^

^*a*^Significant difference (*P* < .05)

IQR indicates interquartile range (quartile 1–quartile 3)

### Perceived impact on preparedness for clinical practice

Following module 1, all students agreed (*n* = 4, 28.6%) or strongly agreed (*n* = 10, 71.4%) that the simulation activity better prepared them to assess and manage a patient with a musculoskeletal disorder. Following module 2, one student (7.1%) was unsure, while the remaining agreed (*n* = 2, 14.3%) or strongly agreed (*n* = 11, 78.6%) that the simulation-based learning activity made them better prepared to manage a patient with a neuromuscular disorder. There was no significant difference between scores across the two modules (*Z* = 0.000, *P* = 1.00).

### Motivation for learning

**Fig. 2:**
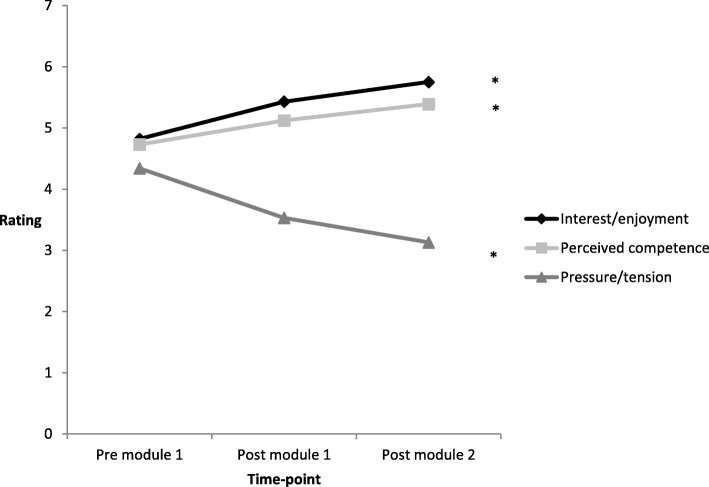
Mean ratings for the Intrinsic Motivation Inventory Subscales across each time point

There was a significant change in students’ interest-enjoyment (*P* < 0.001) across the three time points (pre-module 1, post-module 1, and post-module 2; see Fig. 2). Post hoc tests revealed that the students’ interest-enjoyment following each module was significantly higher than their perceived interest-enjoyment prior to undertaking the simulation-based learning (*P* < 0.001); however, there was no significant difference between the interest-enjoyment following each module. Similarly, there was an overall change in perceived competence across the time points (*P* = 0.036). However, the increase in perceived competence was only significant between pre-module 1 and post-module 2 (*P =* 0*.*029), with no significant change between the other time points. In contrast, perceived pressure and tension significantly decreased over time (*P* = 0.015). The participants reported significantly less pressure-tension following both modules than they expected prior to the simulation-based learning (*P* < 0.05). However, the pressure-tension reported post-modules 1 and 2 were not significantly different.

The perceived usefulness for the development of communication, assessment and management skills was high for all survey points, and there were no significant changes across time for these three measures (Table 4).

**Table 4:** Median (IQR) ratings and results of Friedman’s tests for usefulness subscales on the Intrinsic Motivation Inventory recorded pre-module 1, post-module 1, and post-module 2 (*n* = 14) (where 1 = not at all and 7 = very true)

Usefulness parameter	Module 1		Module 2	Time main effect	
	Pre (IQR)	Post (IQR)	Post (IQR)	*χ*^2^	*P*
Communication skills	7.00 (5.93–7.00)	7.00 (6.43–7.00)	7.00 (6.75–7.00)	2.800	0.247
Assessment skills	7.00 (5.86–7.00)	7.00 (6.00–7.00)	7.00 (6.11–7.00)	3.909	0.142
Management skills	7.00 (5.75–7.00)	7.00 (6.00–7.00)	7.00 (6.22–7.00)	22.70	0.259

IQR indicates interquartile range (quartile 1–quartile 3)

### Perception of the benefits of simulation-based learning

The students’ perceptions of the outcomes and benefits of simulation-based learning were high following each module (medians ranged from 4.00 to 5.00 on the 5-point scale; see Table 4). The majority of participants agreed or strongly agreed to each statement. There were no significant differences in the ratings between the modules.

### Qualitative analysis of perception of most and least effective components of the simulation

Responses to the two post module open-ended questions were grouped into six themes, which are listed, with illustrative quotes in Table 5.

1. Experiential nature of simulation positively impacts learning

**Table 5:** Results of thematic analysis and illustrative quotes regarding the most and least effective aspects of the simulation-based learning

Theme	Participant quotes
(1) Experiential nature of simulation positively impacts learning	“Just being able to go through the process of subjective assessment and put the techniques of listening to practice.” (participant 10)
	“Being able to work with a patient in a simulated setting and to see how well I can communicate” (participant 11)
(2) Debriefing is a valuable component of the simulation-based learning	“Feedback was helpful” (participant 4)
	“Getting feedback from the actor could be beneficial.” (participant 6)
(3) Simulation-based learning is useful for developing interpersonal and communication skills	“Helped to improve my interpersonal skills, as well as aiding to build a better rapport with the patient.” (participant 6)
	“Developing communication skills and note taking ability” (participant 12)
(4) Simulation-based learning is useful for developing history taking	“If anything, trying not to miss any particular parts of the interview was probably the most crucial part, for me.” (participant 2)
	“… practicing the questions asked during an interview” (participant 7)
(5) The realism achieved in the simulation-based learning environment enhances learning	“Being able to observe what a real life situation would be like…” (participant 4)
	“The realistic feel” (participant 13)
(6) Changes to the timing and structure would improve the activity	“The briefing phase of the activity was probably the part that did not impact on the activity. I feel that could be done prior to the task, at home for instance.” (participant 2)
	“Somewhat repetitive and observers are somewhat under-utilised” (participant 13)

Students reported benefit from the opportunity to conduct history taking in the SLE. In module 2, students also valued the opportunity to use the telehealth technology.

(2) Debriefing is a valuable component of the simulation-based learning

Across both modules, students noted that debriefing involving feedback from both the facilitator and SP was the most effective part of the simulation. In some instances, feedback from the SP was not included in the debriefing, which students noted as a limitation.

(3) Simulation-based learning is useful for developing interpersonal and communication skills

A number of students reported that the focus on developing communication and interpersonal skills was most effective.

(4) Simulation-based learning is useful for developing history taking skills

These comments specifically referred to structuring and sequencing history taking, using a range of questioning styles, as well as note taking.

(5) The realism achieved in the simulation-based learning environment enhanced learning

Students reported that the realism created by having a SP, the SLE they portrayed their role in, and the inclusion of co-morbidities in the scenario (module 2) was beneficial.

(6) Changes to the timing and structure would improve the activity

In response to the least effective aspects of the simulation-based learning activity, most students commented on aspects of the timing and structure of the activity which could be improved. These included dedicating less time to briefing prior to the simulation, as it was perceived as repeated information provided in the pre-reading, and making better use of peer observers.

## Discussion

This research evaluated exercise physiology students’ perceptions of practicing history taking in a SLE in terms of their confidence, preparedness for placement, motivation to learn, and perception of benefits. For this cohort of students, the simulation-based learning modules led to an increase in perceived confidence in communication and history taking skills that, in turn, led to students feeling better prepared to manage patients in the pathophysiological areas covered. The students acknowledged the usefulness and benefits of this type of learning and reported that the SLE supported their motivation to learn. These results add to the body of evidence specific to the exercise physiology profession that the use of simulation-based learning and SPs is of value in the education of core clinical skills.

Promoting student confidence is a well-established benefit of learning through SLEs.

[6, 30–32]. This activity was successful in promoting confidence in communication and history taking skills. Although perceived confidence may not be a reliable direct indicator of competence [33, 34], it is acknowledged that it may impact students’ motivation to learn and apply their skills in clinical practice [35, 36]. Positive changes in confidence are important in demonstrating learning has occurred through students building on their prior experiences and gaining new experiences in the simulation-based learning environment to develop self-efficacy and clinical expertise [37]. Previous studies on the use of SPs to educate physiotherapy [16, 34] and exercise physiology [22] students have demonstrated similar gains in confidence in communication and history taking skills. In the present study, confidence continued to rise during the second module, indicating the added value of multiple exposures to the SLE. Having two modules also exposed students to two clinical conditions frequently managed by exercise physiologists. Repetitive practice and exposure to clinical variation are recognized as key aspects of simulation-based learning that promote student learning [38].

The vast majority of students also reported that following the simulation-based learning activity, they felt better prepared to manage a patient with a musculoskeletal or neuromuscular disorder. This finding supports the work by Hecimovich and Volet [21], where preparation for professional practice was a positive theme to emerge from the investigation of exercise physiology students’ perceptions of working with a SP in the management of a patient with a musculoskeletal condition. These findings are important given previous studies have reported a lack of student preparedness for clinical practice [39–41].

Positive primary outcomes in the present study may be partly explained by high levels of motivation. Previous educational research has indicated robust links between motivation and course outcomes [42]. In our study, the change in these measures is in line with the change in confidence, i.e., as confidence increased overtime, there was also a significant increase in interest-enjoyment and perceived competence and a significant decrease in pressure-tension. The pressure-tension scores were mostly mid-range or below (≤ 4 on the 7-point scale) following each module; however, there was a large degree of variability in students’ ratings. It has been suggested that while a moderate level of stress may promote learning, high levels of stress may impair it [43]. It is, therefore, important to ensure a safe, non-threatening learning environment [44]. In the present study, this was achieved by ensuring the students were adequately prepared through the pre-reading and the substantial briefing component of the simulation.

The pre-simulation preparation materials may have also contributed by emphasizing the importance of the simulation-based learning and the objectives of the activities. This may have led to students viewing the simulation-based learning modules as useful for developing clinical skills, as evidenced by the IMI results and the qualitative analyses. However, there was no change in perceived usefulness across time, as students perceived the activity to be useful from the outset. Usefulness or value is linked to motivation through the internalization process, whereby when the learner “sees” an activity as being useful, they will begin to internalize and self-regulate which is a desirable learning outcome [45].

The students identified additional time for preparation and briefing as the least effective aspects of the experience. While preparation and orientation are important to establish rules and expectations for the simulation-based learning activity [6], students perceived that information provided during briefing on the day of the simulation was “repetitive”. This finding is a consideration for future iterations of the simulation-based learning activities which could lead to using different ways to immerse the student in the learning activity. Students did however report that experiential learning, the debriefing, and realism of the patient encounter were effective. The debriefing is recognized as an essential component of the learning experience [38]. Many students in the present investigation reported the value of feedback from multiple perspectives, particularly those of a patient through the SPs. Students who did not receive this feedback noted this as a limitation. Other studies have also reported the usefulness of SPs providing feedback to learners [46, 47].

### Limitations

While we have demonstrated the effectiveness of this simulation-based learning activity in terms of participants’ ratings of confidence and perception of being prepared for practice, we acknowledge there is contention as to whether confidence is associated with skill competency [30, 48, 49]. It would, therefore, be beneficial in the future to include behavioral measures of skill attainment/competency. As students completed questionnaires immediately following the modules, it is unclear whether the perceptions of increased confidence continued to their clinical practicum. Future studies should include evaluation of students during their clinical practicum to establish the sustainability of changes in student skill and effective translation of skills into the placement setting. The largely novice nature of the student participants in relation to their experience with simulation-based learning could have been a contributing factor to the positive responses observed in this study. This study was conducted on a relatively small number of student participants from one institution, thus limiting the generalizability of the findings. The inclusion of additional student cohorts and an expansion to include students from other institutions could be a future research direction.

## Conclusions

The results of this study indicated that simulation-based learning with SPs increased the confidence and perceived preparedness of exercise physiology students for conducting history taking, a requisite exercise physiology skill. High student motivation and the core features of quality simulation-based learning activities (experiential learning, realism, and debriefing) contributed to student perceptions of the value of this activity.

## Additional file

Additional file 1: Student Workbook. (DOCX 11 kb)

## Abbreviations

CI: Correlation index
IMI: Intrinsic motivation inventory
IQR: Interquartile range
SD: Standard deviation
SLE: Simulated learning environment
SP: Simulated patient
