# Supplementary material for: Simulated patient contributions to enhancing exercise physiology student clinical assessment skills
Source: Adv Simul (Lond). 2019 Dec 20;4(Suppl 1):15. doi: 10.1186/s41077-019-0097-6 (PMC6923845; doi:10.1186/s41077-019-0097-6)
Supplement: Supplementary file 1 — Student Workbook. (DOCX 11 kb) [file 41077_2019_97_MOESM1_ESM.docx]

**Appendix 1.** Student Workbook

**Briefing**

Basic Principles of a Patient Interview

What strategies will you use to develop a positive report with your patient?

How will you ensure that your patient assessment remains ‘patient-centred’?

How will you open/begin the patient assessment?

What strategies will you employ to ensure that you obtain a full and detailed background on the patient’s presenting concerns?

Interview Sequence

Describe the interview sequence you will use during the patient assessment?

**Debriefing**

What aspects of the patient assessment went well?

What aspects of the patient assessment could be improved?

What strategies were used during the patient assessment to develop a report with the patient and how effective were they?

What strategies were used to keep the patient assessment ‘patient-centred’ and how effective were they?

What strategies were used to obtain a full and detailed background on the patient’s presenting concerns and how effective were they?
